# A decade of Tropical Legumes projects: Development and adoption of improved varieties, creation of market‐demand to benefit smallholder farmers and empowerment of national programmes in sub‐Saharan Africa and South Asia

**DOI:** 10.1111/pbr.12744

**Published:** 2019-08-14

**Authors:** Rajeev K. Varshney, Chris Ojiewo, Emmanuel Monyo

**Affiliations:** ^1^ International Crops Research Institute for the Semi‐Arid Tropics (ICRISAT) Patancheru India; ^2^ International Crops Research Institute for the Semi‐Arid Tropics (ICRISAT) Nairobi Kenya

**Keywords:** breeding, food security, genomics, legumes, seed system

## Abstract

This article highlights 12 years (2007–2019) of research, achievements, lessons learned, challenges and gaps in discovery‐to‐delivery research in legumes emanating from three projects, collectively called Tropical Legumes (TL) with a total investment of about US$ 67 million funded by the Bill & Melinda Gates Foundation. These projects were implemented by three CGIAR centres (ICRISAT, CIAT and IITA) together with 15 national agricultural research system partners in sub‐Saharan Africa and South Asia. The TL projects together with some of their precursors and complementary projects from other agencies, facilitated the development of 266 improved legume varieties and the production of about 497,901 tons of certified seeds of the target legume crops in the focus countries. The certified seeds have been planted on about 5.0 million ha by more than 25 million smallholder farmers in the 15 countries and beyond, producing about 6.1 million tons of grain worth US$ 3.2 billion. Furthermore, the projects also trained 52 next generation scientists that included 10 women, by supporting 34 Masters degrees and 18 PhD degrees.

## 1 INTRODUCTION

**Figure 1:**
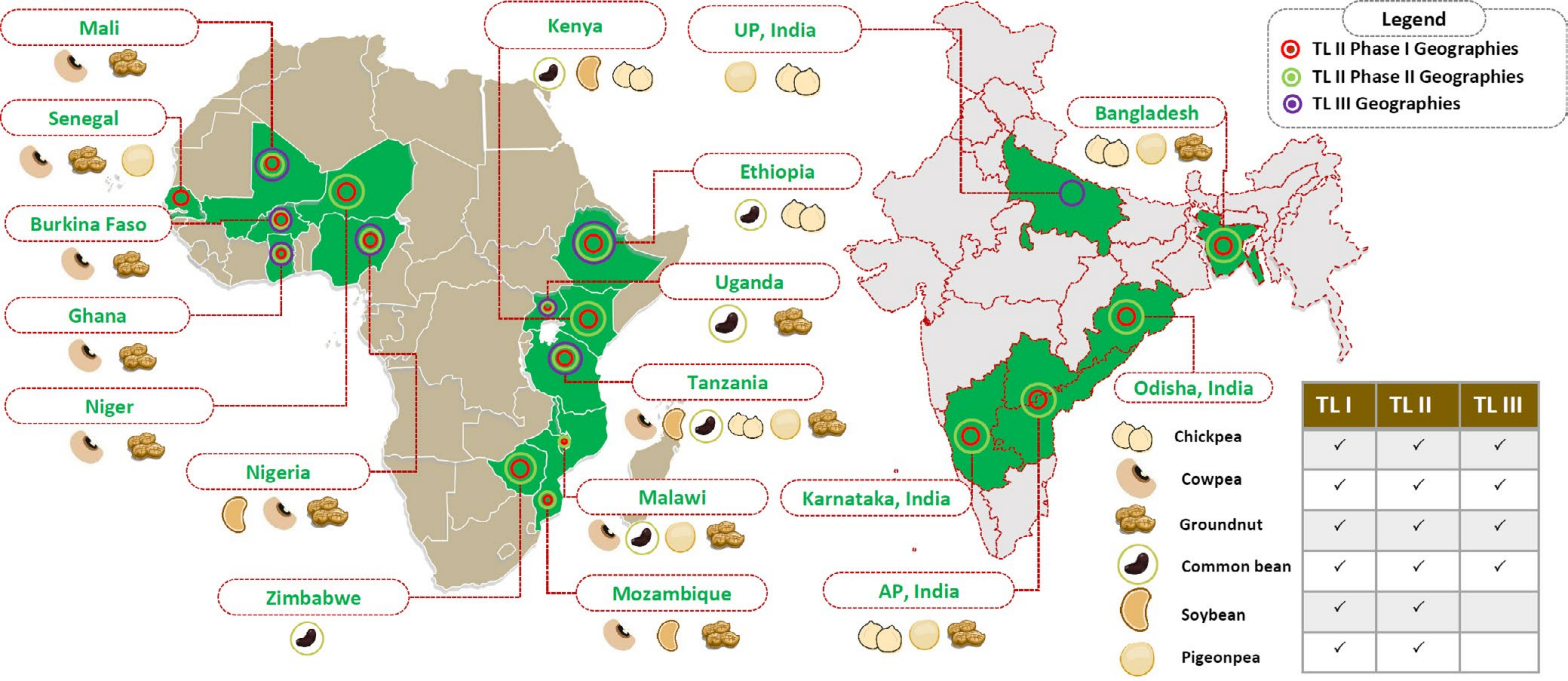
Tropical Legumes (TL) crops and geographies. Phases I and II of TL II projects conducted research and development activities on six legume crops, namely chickpea, common bean, cowpea, groundnut, pigeonpea and soybean. The TL III project focused on four crops, i.e., chickpea, common bean, cowpea and groundnut. In terms of geographies, TL II phase I had project activities in 15 countries, namely Burkina Faso, Ghana, Mali, Niger, Nigeria, Senegal, Ethiopia, Kenya, Malawi, Mozambique, Tanzania, Uganda and Zimbabwe in sub‐Saharan Africa (SSA) and India and Bangladesh in South Asia (SA), while phase II involved all the above mentioned countries except Senegal. TL III focused on seven countries in SSA (Burkina Faso, Ghana, Mali, Nigeria, Ethiopia, Tanzania and Uganda) and the Uttar Pradesh state of India in SA [Colour figure can be viewed at http://www.wileyonlinelibrary.com]

The Tropical Legumes (TL) projects supported by the Bill & Melinda Gates Foundation (BMGF) were jointly implemented by three CGIAR Centres (ICRISAT, CIAT and IITA) together with 15 national agricultural research system (NARS) partners in select focus countries (Figure 1). The projects ran in three phases: – TL II Phase I (2007–2011, US$ 20.603 million), TL II Phase II (2012–2014, US$ 21.420 million) and Phase III or TL III (2015–2019, US$ 24.970 million). The projects aimed to improve the livelihoods of smallholder farmers in drought‐prone areas of Sub‐Saharan Africa (SSA) and South Asia (SA) through improved productivity and production of six major grain legume crops – chickpea (*Cicer arietinum*), common bean (*Phaseolus vulgaris*), cowpea (*Vigna unguiculata*), groundnut (*Arachis hypogaea*), pigeonpea (*Cajanus cajan*) and soybean (*Glycine max*). The project activities were implemented in Burkina Faso, Ghana, Mali, Niger, Nigeria, Senegal, Ethiopia, Kenya, Malawi, Mozambique, Tanzania, Uganda and Zimbabwe in SSA and India and Bangladesh in SA (Figure 1).

While Phases I and II of the project focussed on research and development activities in six target legume crops, Senegal was the only country that was dropped in Phase II. TL III strategically focused on fewer crops (chickpea, common bean, cowpea and groundnut) and fewer geographies (Burkina Faso, Ghana, Mali, Nigeria, Ethiopia, Tanzania and Uganda; and Uttar Pradesh state of India). It also aimed to fundamentally strengthen the NARS and CGIAR's breeding programmes and seed platforms to enhance their ability to deliver high and sustained technology outputs to smallholder farmers. Capacity building remained an important component of the three phases.

This special issue of Plant Breeding showcases the efforts and advances made in the six legume crops through TL and other associated projects. The issue includes eight reviews and perspective articles from leading researchers engaged in the TL projects and an invited article on faba bean. The issue highlights the advances made in the areas of genetics, genomics and deployment of integrated breeding approaches for developing improved drought‐ and heat‐ tolerant and biotic stress‐resistant varieties in the target legume crops. Furthermore, market‐led options to scale up legume seeds in developing countries have also been presented in an article. A snapshot of these articles as well as a summary and perspectives from the Guest Editors including Rajeev K. Varshney, the Principal Investigator (2013–2019); Chris Ojiewo, Project Coordinator (2017–2019) and Emmanuel Monyo, Project Coordinator (2012–2016) are presented in this article.

## 2 CHICKPEA ‐ ADAPTATION TO DROUGHT AND HEAT STRESSES

In the face of diminishing rainfall and increasing temperatures globally, developing drought‐ and heat‐tolerant chickpea varieties becomes imperative considering that chickpea is one of the important legume crops largely grown by smallholder farmers who are more susceptible to climate variability and change. Therefore, efforts were made to develop several early‐maturing (drought escaping) varieties with improved drought tolerance by selecting for grain yield under moisture stress conditions. Similarly, selection for pod set in the crop subjected to heat stress during the reproductive stage has helped in developing heat‐tolerant varieties. In parallel, significant advances were made in decoding the chickpea genome (Varshney, Song, et al., 2013), sequencing several hundred germplasm lines and varieties (Varshney, Thudi, et al., 2019), developing genetic and physical maps and identifying quantitative trait loci (QTLs) including “*QTL‐hotspot*” region containing QTLs for several drought tolerance traits. Some of these efforts were partially supported by TL projects as well as the CGIAR Generation Challenge Programme. In the TL projects, the “*QTL‐hotspot*” region was also introgressed into several popular cultivars using marker‐assisted backcrossing (MABC) and introgression lines giving significantly higher yield than the popular cultivars (Thudi et al., 2014; Varshney, Gaur, et al., 2013). Similarly, molecular breeding has been used successfully to develop resistant lines for Fusarium wilt and ascochyta blight resistance (Mannur et al., 2019; Pratap et al., 2017; Varshney, Mohan, et al., 2014). During the three phases of the project, 28 chickpea varieties were developed and released and 259,552 tons of certified seeds of modern improved varieties were produced and planted on about 2.2 million ha (Table 1). Some of these achievements have been presented in detail by Gaur et al. (2019) in this issue.

**Table 1:** Varieties released, seed production and area covered under TL projects (2007–2017) with contributions and the support of precursor and complementary projects

Crop	Tropical Legume (TL) Phases	Country	Varieties developed	Seeds produced (tons)	Area under new varieties (ha)
Chickpea	TL II Phases I & II	Ethiopia	8	158,471	1,320,592
		Tanzania	4	1,412	11,767
		Kenya	9	1,461	12,174
		India	3	211,411	1,761,762
		Bangladesh	1	137	1,145
	TL III	Ethiopia	3	8,951	74,589
		India	0	725	6,042
		Total	28	382,569	3,188,071
Cowpea	TL II Phases I & II	Burkina Faso	6	0	0
		Ghana	4	0	0
		Mali	2	751	18,775
		Nigeria	7	3,712	92,788
		Senegal	0	0	0
		Niger	5	6,146	153,640
		Mozambique	0	774	19,338
		Tanzania	0	2	58
	TL III	Burkina Faso	0	1,353	33,829
		Ghana	0	2,342	58,548
		Mali	5	1,484	37,102
		Nigeria	0	3,790	94,750
		Total	29	20,353	508,825
Common bean	TL II Phases I & II	Ethiopia	15	27,702	277,021
		Kenya	17	5,657	56,568
		Malawi	13	3,216	32,156
		Tanzania	12	2,268	22,683
		Uganda	18	15,183	151,834
		Zimbabwe	7	1,373	13,729
		Nigeria	0	0	0
	TLIII	Ethiopia	13	13,575	135,745
		Tanzania	9	3,821	38,214
		Uganda	0	23,735	237,353
		Total	104	96,530	965,302
Groundnut	TL II Phases I & II	Burkina Faso	0	613	6,130
		Ghana	0	417	4,169
		India	16	20,092	200,916
		Bangladesh	0	166	1,660
		Mali	9	1,380	13,798
		Nigeria	3	2,562	25,619
		Senegal	6	3	33
		Niger	5	1,420	14,196
		Tanzania	9	25,455	254,550
		Uganda	12	578	5,777
		Malawi	7	18,431	184,311
		Mozambique	6	133	1,327
	TL III	Burkina Faso	0	7,944	79,440
		Ghana	0	205	2,051
		Mali	0	2,929	29,294
		Nigeria	0	7,843	78,425
		Tanzania	3	7,416	74,155
		Uganda	0	4,049	40,488
		Total	76	101,634	1,016,339
Pigeonpea	TL II Phases I & II	Ethiopia	0		0
		India	7	2,371	94,854
		Bangladesh	0	0	0
		Kenya	3		0
		Tanzania	0	1,488	59,536
		Uganda	0	39	1,572
		Zimbabwe	0		0
		Mozambique	4		0
		Malawi	3	1,787	71,488
		Zambia	2	0	0
		Total	19	5,686	227,450
Soybean	TL II Phases I & II	Ethiopia	0		0
		Kenya	7	341	4,541
		Tanzania	2		0
		Uganda	0		0
		Zimbabwe	0		0
		Mozambique	0	4,823	64,305
		Malawi	0	122	1,627
		Nigeria	1	8,860	118,134
		Total	10	14,146	188,607
	Total		266	620,917	6,093,267

## 3 COMMON BEAN – MARKET‐DRIVEN BREEDING AND GENDER‐RESPONSIVE PARTICIPATORY VARIETAL SELECTION

Guided by market‐driven approaches to develop client preferred common bean varieties, significant efforts were made to address the production constraints and develop multi‐trait common bean varieties. In parallel, the genome sequence of common bean became available from a US‐led consortium (Schmutz et al., 2010). Molecular markers for several traits were developed (Mukankusi‐Mugisha et al., 2019) and used for selection in breeding programmes to develop varieties resistant to key diseases and insect pests. In brief, 104 common bean varieties were developed and released and 96,530 tons of certified seed were produced and planted on about 965,302 ha (Table 1). Some of these achievements have been presented by Mukankusi‐Mugisha et al. (2019). The authors have also highlighted the efforts to utilize modern genomic tools to increase scale, efficiency, accuracy and speed of breeding. In addition, adoption of gender‐responsive participatory variety selection that led to the release of several market preferred varieties in 31 African countries has been highlighted.

## 4 COWPEA – A BETTER UNDERSTANDING OF GENETICS, GENOMICS AND DEPLOYMENT OF MODERN BREEDING APPROACHES

Cowpea is an important source of protein for millions of smallholder farmers, mainly in West and Central Africa. However, abiotic and biotic stresses adversely affect its productivity and production. Efforts under TL projects led to a better understanding of genetics, genomics and breeding of cowpea and various abiotic and biotic factors affecting its yield. TL, together with other sister projects, contributed to the development of cowpea genomic resources such as consensus genetic map (Lucas et al., 2011), genome sequence (Lonardi et al., 2019), etc. The cowpea team in TL projects aimed at developing drought tolerant, phosphorus use efficient, bacterial blight and virus resistant lines by exploiting available genetic resources and deploying modern breeding tools. Through the support of the TL projects, 29 cowpea varieties were developed and 20,353 tons of certified seeds were produced and planted on about 508,825 ha (Table 1). These efforts have been highlighted by Boukar et al. (2019) in this special issue.

## 5 GROUNDNUT – ADVANCES IN GENOMICS AND IMPLEMENTATION OF INTEGRATED BREEDING APPROACHES

Low groundnut yields in Asia and Africa can be attributed to various production constraints, leading to less production and low income for smallholder farmers. Advances made under TL and associated projects led to a better understanding of the groundnut genome, discovery of genes/variants for traits of interest and the integration of marker‐assisted breeding for selected traits. Under TL projects, a total of 76 groundnut varieties were developed and released and 101,634 tons of certified seed were produced and planted on about 1 million ha by 2017 (Table 1). The integration of genomic tools in the breeding process accompanied by increased precision of yield trialling and phenotyping is expected to increase efficiency and enhance genetic gain for the released improved groundnut varieties. There are already several success stories of development of improved lines for resistance to foliar diseases (Pasupuleti et al., 2016; Varshney, Pandey, et al., 2014) and for high oleic acid content (Bera et al., 2018; Janila et al., 2016). In the review paper by Desmae et al. (2019), the authors highlight the advances in genetics, genomics and breeding to improve the productivity of groundnut. Recent availability of genome sequences of tetraploid groundnut (Bertioli et al., 2019; Chen et al., 2019; Zhuang et al., 2019) will help in further accelerating molecular breeding in groundnut.

## 6 PIGEONPEA ‐ AN AMALGAM OF BREEDING AND GENOMIC RESEARCH

Pigeonpea was part of the legume crops portfolio in two phases of the project (TL II Phases I and II), during which 19 improved pigeonpea varieties and hybrids were developed and released and 5,686 tons of certified seed were produced and planted on about 227,450 ha (Table 1). With partial support from this project and others, significant advances were made in genomics research. Availability of the draft genome sequence (Varshney et al., 2012) with large‐scale marker resources (Chanda Venkata et al., 2018) oriented research towards trait mapping for flowering time, determinacy, fertility restoration, yield attributing traits and photo insensitivity. Molecular markers have been developed for assessing the purity of hybrids (Bohra et al., 2011; Saxena, Saxena, & Varshney, 2010). Modern genomic tools such as next‐generation sequencing and genome‐wide selection are leading towards next generation breeding and enhancing selection efficiency. The paper by Chanda Venkata et al. (2018) emphasizes the ongoing genetic improvement in pigeonpea that integrates conventional breeding with genomic research.

## 7 SOYBEAN ‐ PUBLIC SECTOR BREEDING FOR CULTIVAR DEVELOPMENT IN THE AFRICAN TROPICS

Cultivated soybean is the number one oil and protein supplier for animal and human nutrition. It accounts for about 84.5% of the world's grain legumes trade (Abate et al., 2011). Although SSA accounts for less than 2% of the global production, it constitutes an important component in smallholder cropping systems. In SSA, soybean has made comparable contribution to the growth in production with annual growth rates of 3.0% in area and 3.5% in yield. Despite these positive trends, average soybean yields in Africa (1.2 tons/ha) are much lower than the global average of about 2.5 tons/ha. The low yields are due to a number of production constraints including low adoption of improved varieties and poor agronomic practices. Two phases of TL projects, together with other associated projects, facilitated the development and release of 10 improved varieties and the production of 14,146 tons of certified seeds that replaced old varieties on about 188,607 ha (Table 1). Chigeza et al. (2019) provide an overview of current soybean breeding in SSA and an update on the accomplishments of the IITA soybean breeding programme.

## 8 FABA BEAN ‐ BREEDING FOR BIOTIC AND ABIOTIC STRESSES

Although faba bean (*Vicia faba*) was not part of the TL projects, we included an article on advances in genetics and breeding of faba bean by Maalouf et al. (2018) as this is an important grain legume crop in Africa, especially because of its high yield potential and nutrition‐dense grains. This article presents efforts and achievements made in faba bean improvement in the last four decades, which led to the doubling of global average yields. The authors have reviewed the genetic diversity, breeding methodologies, major achievements in biotic and abiotic traits and recent molecular approaches. Maalouf et al. (2018) anticipate the development of more coherent genetic maps to facilitate the assembling and ordering of genomic scaffolds in future efforts of genome sequencing, trait discovery and molecular breeding approaches.

## 9 MARKET‐LED OPTIONS TO SCALE UP LEGUME SEEDS IN DEVELOPING COUNTRIES

Smallholder farmers need not only improved varieties, but also connection to markets so that they can generate more income. Therefore, TL projects invested in this direction as well. It fostered innovative public‐private partnerships in joint testing of innovative market‐led seed systems, skills and knowledge enhancement and de‐risking private sector initiatives by introducing new approaches that were previously overlooked in technology delivery. As new public and private seed companies, individual seed entrepreneurs and farmer organizations emerged, the capacities of existing ones were enhanced. This resulted in significant rise in production, availability and accessibility of various seed classes and grades of newly improved and farmer preferred legume varieties in the target countries. These experiences have been documented by Rubyogo et al. (2019) in this issue.

## 10 INTEGRATION OF GENOMICS, GENETICS, BREEDING AND SEED SYSTEMS TO ACCELERATE GENETIC GAINS

The article by Ojiewo et al. (2019) highlights the importance of continuous genetic improvement for enhanced productivity, production, quality and adoption of higher yielding cultivars to enhance their sustainable and timely availability, accessibility and affordability. It discusses exploring of plant genetic resources and their genetic characterization, trait discovery based on genome sequences and large‐scale marker resources available and pre‐breeding approaches. The authors also highlight the value of diagnostic markers for early generation selection and molecular breeding by providing the current status of their availability and usage. A detailed account is given of the deployment of molecular breeding for developing superior lines. Finally, the authors provide a road map to develop better varieties rapidly by integrating different genomic, genetic and breeding approaches. Adoption of decision support tools may help achieve greater scale (Varshney et al., 2016). In addition, improved cultivars of the legume crops are also more responsive to improved crop management for high productivity, making them increasingly more relevant to reducing hunger in the areas they are traditionally grown and consumed.

## 11 IMPACT ON THE GROUND

During Phases I and II of the project, disseminated improved varieties were adopted on at least 4.0 million hectares and more than US$ 2.6 billion was generated from the project and investment partners. This is far above the total TL II (Phase I and Phase II) investment grossly compounded at US$ 48 million. Leveraging direct and partner investments, the project generated US$ 54 for each dollar invested during TL II. Furthermore, TL III supported the production of 90,161 tons of certified/QDS seed in its first 3 years (2015–2017). This seed was planted on about 1 million ha to produce 1.2 million tons of grain valued at almost US$ 616 million. This implies that for every US$ 1 invested in TL projects, there is a gross return of about US$ 25 (Monyo & Varshney, 2016). The TL III project alone has reached about 5 million smallholder legume farmers with high quality seed of improved varieties at an average landholding of 0.2 ha/household under promoted legumes. Overall, the BMGF investment in TL II and TL III projects has supported the production of about 498,000 tons of certified seeds for the target legume crops over the past decade (2007–2017) in SSA and South Asia. This certified seed has been planted on about 5.0 million ha by more than 25 million smallholder farmers in 15 countries and produced about 6.1 million tons of grain worth US$ 3.2 billion (Table 1).

Impact for change studies were conducted in all Tier 1 countries and crops – Nigeria (groundnut and cowpea), Tanzania (common bean and groundnut), and Ethiopia (common bean and chickpea). An interesting example of common bean adoption in southern Tanzania involves variety 'Uyole 96' that had been dominating more than 60% of production by area. By 2016, 'Uyole 96' had been replaced by TL project‐promoted 'Njano Uyole' variety that is appreciated for its agronomy, consumption, processing and marketing attributes such as better yield, pest/disease resistance, ease of shelling, storability, market price, colour and ease of cooking.

The technological progress in the form of varietal change and improved agronomic practices have combined to provide positive growth trends in common bean productivity in Ethiopia. For example, yield grew from 1.0 ton/ha in 2008 to about 1.7 ton/ha in 2016. After accounting for cofounding factors, the adoption of improved common bean varieties increased the average yield of beans by 0.336 tons/ha. The national adoption rate of improved varieties in Ethiopia is about 37% of bean growers, which translates to about 1.5 million households. 'Mexican 142' variety that was controlling over 50% of the white canning bean market class and 'Red Wolita' variety that was controlling about 70% of the red cooking bean type at the time of baseline studies in 2009 have been totally replaced by new varieties promoted under the project.

Improved groundnut varieties in Nigeria registered an overall adoption rate of 44% (60% for females and 42% for males) by sampled farmers. When the project started in 2007, old varieties released between the 1960s and early 1990s, such as 'Samnut 1' to 'Samnut 20', were still dominant. The TL projects promoted the adoption of 'Samnut 21', Samnut 22' and 'Samnut 23' released in 2000 before the release of newer varieties 'Samnut 24' (2011), 'Samnut 25' (2013) and 'Samnut 26' (2013) which have replaced these old varieties. 'Samnut 24' currently controls about 25% of groundnut production. The project's interventions and enabling factors, including strategic partnerships, led to a significant yield increase of 0.222 tons/ha (0.391 tons/ha for females and 0.200 tons/ha for males) and associated income increase of US$ 135/ha (US$ 168/ha for females and US$ 93/ha for males).

In Nigeria, old varieties of cowpea such as 'Sampea 7', released in 1985, have almost been completely replaced by newer varieties such as 'Sampea 8' released in 2005 and promoted under the project since 2007. Better still, 'Sampea 8' was quickly replaced by 'Sampea 11' released in 2009, which in turn is currently seeing a swift replacement by 'Sampea 14' and 'Sampea 15' (released in 2011), 'Sampea 16' and 'Sampea 17' (released in 2015) and Sampea 18 and Sampea 19 (released in 2018).

The latest data on chickpea varietal adoption at the national level in Ethiopia are awaiting analysis. However, an earlier study conducted in three intervention districts showed up to 80% adoption levels of new varieties although the national average is estimated to be less than 30% (Verkaart, Munyua, Mausch, & Michler, 2017). Together with integrated crop management practices, chickpea productivity increased from 1.27 tons/ha in 2007 to 1.97 tons/ha in 2016; and total national production rose from 253,871 tons on about 200,066 ha in 2007 to 444,146 tons on about 225,608 ha in 2016. The change in production is about 75% over 2007 base figures, mainly accounted for by gain in productivity (55%) rather than area (13%).

On the other hand, in Tanzania, adoption of improved groundnut varieties is estimated at 19% nationally before correction through DNA fingerprinting data. However, the seed system works through about 400 farmer research groups linked to seed companies as contract seed producers, together with training and the adoption of integrated crop management practices have contributed to increased groundnut productivity from 0.724 tons/ha in 2008 to about 1.010 tons/ha in 2015 and total production from 340,770 tons on about 470,670 ha to 1,635,335 tons on about 1,619,500 ha in 2015. The change in production is about 480% over 2008 base figures and both gains in area (244%) and productivity (39%) have contributed to these remarkable increases. The varieties that were reigning before 2007 include 'Nyota' (1.5 tons/ha), 'Johari' (1.0 tons/ha), 'Sawia' (1.5 tons/ha) and 'Pendo' (1.5 tons/ha). While 'Pendo' is still dominant and is currently being replaced by rosette‐resistant 'Nachingwea' (1.0–1.5 tons/ha) and 'Mangaka' (1.5–1.8 tons/ha), the other varieties have largely been replaced.

In Mali, the ruling groundnut varieties before 2007 were very old, some dating back to 1928. New varieties less than 10 years old were promoted by the project. 'Fleur 11' and 'ICGV 86124' are currently replacing the old varieties because of their high fodder yield and short duration in an area with rainfall shortage where most farmers prefer early‐maturing varieties with high pod and haulm yields for livestock.

Finally, for sustainable legume production, it is not enough to generate new high yielding market preferred varieties and seed production; instead, it is equally important to have a well trained next generation of breeders. With this objective, TL projects have trained 34 Masters degree and 18 PhD degree students including 10 females and 42 males (Table 2).

**Table 2:** Number of students trained under TL projects

Country	Degree	Crop	Name	Sex	Male	Female	Total
Burkina Faso	PhD	Cowpea	Gnankambary/Trore Karidiatou	F	1	1	2
	PhD	Cowpea	Lalsaga Joel	M			
Ethiopia	MSc	Chickpea	Mitiku Demissie	M	3		3
	MSc	Chickpea	Mekbib Gebretsadik	M			
	MSc	Chickpea	Tadesse Sefera	M			
	PhD	Common bean	Teshale Assefa	M	3		3
	PhD	Common bean	Berhanu Amsalu	M			
	PhD	Common bean	Kidane Tumsa Hurisa	M			
Ghana	MSc	Groundnut	Wohor Zakaria Osman	M	1	1	2
	MSc	Cowpea	Grace Adusei	F			
	PhD	Cowpea	Haruna Mohammed	M	1		1
India	MSc	Pigeonpea	Viskas Navhale	M	1		1
	PhD	Chickpea	Tosh Garg	M	3		3
	PhD	Pigeonpea	Rachit K. Saxena	M			
	PhD	Pigeonpea	S. L. Swargaonkar	M			
Kenya	MSc	Chickpea	Peter Kaloki	M	2	2	4
	MSc	Common bean	Waweru Felix Muchiri	M			
	MSc	Chickpea	Nancy W. Njogu	F			
	MSc	Seed systems	Scolastica Wambwa	F			
	PhD	Agro‐enterprise	David Nyongesa	M	1		1
Malawi	MSc	Groundnut	Wilson Chafutsa	M	1		1
	PhD	Common bean	Lizzie Kachulu	F		1	1
Mali	MSc	Cowpea	Siaka Dembele	M	4		4
	MSc	Ag economics	Abdoulaye Diarra	M			
	MSc	Groundnut	Mamary Traore	M			
	MSc	Cowpea	Diarra Youssouf	M			
	PhD	Cowpea	Ibrahima Z. Doumbia	M	1		1
Mozambique	MSc	Cowpea	Guilhermino Boina	M	3	1	4
	MSc	Cowpea	Henrique Victor Colial	M			
	MSc	Cowpea	John Bulassi Kaunda	M			
	MSc	Soybean	Anica S.F. Massas	F			
Niger	MSc	Groundnut	Nana M.1. Garba	F	1	1	2
	MSc	Cowpea	Abdou Souleymane	M			
Nigeria	MSc	Cowpea	Habibu Aliyu	M	7		7
	MSc	Soybean	Shaahu Aondover	M			
	MSc	Cowpea	Auwal Adamu Umar	M			
	MSc	Cowpea	AK Olomide Oluwatosin	M			
	MSc	Cowpea	Oluwaseyi Toyinbo				
	MSc	Cowpea	Jonathan Joseph Iduh Otene	M			
	MSc	Groundnut	Shiyanbola Abiodun Abdulsalam	M			
	PhD	Cowpea	Kayode Ogunsola	M	2	1	3
	PhD	Cowpea	Oladejo Samuel Atanda	M			
	PhD	Groundnut	Kalat Patience Duniya	F			
Tanzania	MSc	Groundnut	Mohamed Ismael	M	6		6
	MSc	Cowpea	Didasi R. Kimaro	M			
	MSc	Groundnut	Juma Mfaume	M			
	MSc	Cowpea	Julius Missanga	M			
	MSc	Soybean	Justine Alfred Mushi	M			
	MSc	Common bean	Julius Peter Mbiu	M			
	PhD	Pigeonpea	Maryama Mayomba	F		2	2
	PhD	Groundnut	Happy Makuru Daudi	F			
Zimbabwe	PhD	Common bean	Godwill Makunde	M	1		1
Total					42	10	52

## 12 SUMMARY AND OUTLOOK

TL projects helped the CGIAR and their NARS partners develop and release 266 improved varieties of targeted legume crops, produce about 498,000 tons of certified seeds of the legume crops in the target geographies and train 52 new scientists. TL and other related projects also helped to develop genomic information, diagnostic markers and deploy them in breeding programmes. Some of these results have been compiled by Ojiewo et al. (2019) in the special issue. Legume breeding in SSA and SA (India) has come of age through the investment of TL and other related projects. The time has come to turn to sequence‐based breeding in these legume crops. Furthermore, while the integration of genomic information and deployment of modern breeding approaches such as sequence‐assisted breeding (Varshney, Pandey, et al., 2019) and speed breeding (Watson et al., 2018) can accelerate the development of superior varieties, it is crucial to have a strong seed delivery system in SSA and SA so that farmers can have access to improved varieties (Varshney et al., 2018). The pace at which old varieties are replaced by new ones needs to be accelerated. Adopting appropriate agronomic practices while cultivating improved varieties will help in realizing the full potential of genetics and breeding and in delivering more produce to farmers. At the same time, providing farmers access to markets will fetch them more income to improve their livelihoods. Subsequently, market feedback should go back in the loop to define traits in the development of market‐led and climate resilient varieties. The future of legumes breeding is bright and promises to benefit smallholder farmers in SSA and SA.

## CONFLICT OF INTEREST

The authors declare that they have no conflicts of interest.

## AUTHORS’ CONTRIBUTION

RKV as Principal Investigator and CO as the Coordinator for the Tropical Legumes III and EM as the former Coordinator for the Tropical Legumes III and II, have compiled achievements of Tropical Legumes presented in this article. RKV wrote the first draft of the article and finalized with contributions of CO and EM.
